# Trends in sensorimotor research and countermeasures for exploration-class space flights

**DOI:** 10.3389/fnsys.2015.00115

**Published:** 2015-08-11

**Authors:** Mark Shelhamer

**Affiliations:** NASA Human Research Program, NASA Johnson Space CenterHouston, TX, USA

**Keywords:** spaceflight, adaptation, sensorimotor, vestibular, integrative physiology

## Abstract

Research in the area of sensorimotor and neurovestibular function has played an important role in enabling human space flight. This role, however, is changing. One of the key aspects of sensorimotor function relevant to this role will build on its widespread connections with other physiological and psychological systems in the body. The firm knowledge base in this area can provide a strong platform to explore these interactions, which can also provide for the development of effective and efficient countermeasures to the deleterious effects of space flight.

A long-term goal of many of the major space programs is to send people to Mars, or other similarly challenging destinations far from the relative safety of low earth orbit. Such a mission would entail an extended journey, both spatially and temporally. Some of the many hazards of this type of journey include isolation, confinement, remoteness, radiation, and extended weightlessness (Buckey, 2006; Piantadosi, 2012). All of these hazards can have effects on different aspects of sensorimotor (including neurovestibular) function. This is in part because sensorimotor processes, being intimately connected to the central nervous system, have widespread interactions with other physiological and psychological processes. By the same reasoning, it is important to understand these interactions so that more effective multi-disciplinary countermeasures and interventions might be designed. The sensorimotor system has an almost unique position in being the hub of most other bodily processes. Thus it is important not only to develop countermeasures for the classical problems of sensorimotor function—motion sickness, disorientation, ataxia—but also to understand the connections to other systems so that integrated countermeasures might be developed. The author’s position in the NASA Human Research Program provides a unique perspective on the countermeasures being developed for various physiological deficits, their interactions and potential synergies, and the operational concerns relevant to their implementation, which informs the presentation here.

## Overview of Sensorimotor Effects of Space Flight

A large amount of high-quality research has been conducted in the general area of sensorimotor and neurovestibular responses to extreme environments over the past decades, spurred in part by the problems of human functioning in high-performance aircraft, and problems encountered in the early days of human space flight (Reschke et al., 1998; Paloski et al., 2008).

A primary driver of sensorimotor disturbances in space flight is the lack of tonic otolith stimulation due to the lack of a constant g-vector. On earth, the nervous system expects this tonic signal, as transduced predominantly by the otolith organs, and incorporates it into the programming of vestibularly mediated movements and perceptions. Upon entering the spaceflight environment, the nervous system must adapt to the loss of this signal, during which motion sickness, disorientation, and movement disturbances (such as oscillopsia due to miscalibrated vestibular reflexes) may occur. Overt adaptation typically is accomplished within three days. It should be noted that this adaptive process is appropriate for the environment: the nervous system learns not to expect a static g-vector and the attendant tonic otolith signal, and adjusts accordingly. Upon return to earth (or another location with a static g field), however, the space-appropriate adaptation is now inappropriate, and similar problems again occur during this transition and readaptation stage. Especially troubling is that the full readaptation process can take weeks or months (depending on how long the person has been in space), the initial exposure to a g field can be extremely dynamic (due to vehicle maneuvers), and the crew will be called on to perform demanding tasks related to reentry and return during this time. Related to these disturbances are well-documented problems with posture and locomotion upon return to earth, due to the vestibular processes just described and to the fact that the usual processes of locomotion are not called into play during the weightlessness of space flight. These particular disturbances have an impact on the ability to leave the spacecraft, either in an emergency situation or for planned exploratory ventures. While there are many other changes which occur with varying degrees of intensity (Clément and Reschke, 2008), these are the primary ones that are relevant to the discussion that follows, and the ones most likely to be of consequence for long-duration exploration space flights.

Thus we understand a great deal about the effects of space flight on sensorimotor function, especially neurovestibular issues. Key problems are expected during g transitions due to the need to apply sensorimotor programs acquired in one g level, in a new and changing g level (Black et al., 1999; Harm et al., 1999); although there are issues involving spatial orientation that are longer term. Some of these problems can be addressed with crew training and automation, as well as operational concepts. As an example, after a landing on Mars with a deconditioned crew, it may be possible to shelter in place and stand down for a week before undertaking extravehicular activities (EVAs) and other exploratory activities. An appropriate mission design can take this need into account, if it is determined that maintaining high levels of crew sensorimotor function immediately on landing is unrealistic or infeasible given the resources available on the journey. Of course there may well be sensorimotor concerns during the landing itself, even if it is highly interactive and aided by automation. However, if the countermeasure to alleviate the landing concerns is expensive in terms of resources then automation will be relied upon, with the crew in a monitoring role. There might be benefit here from research efforts that determine individual adaptive capacity which can help in assigning crew roles (Noohi et al., 2014; Wong and Shelhamer, 2014).

## Risk Assessment and Mitigation

It is useful to keep in mind that sensorimotor effects of space flight are different from many others tracked by NASA. One broken bone might disable an entire crew: it is long-lasting and requires significant care. One person with motion sickness will not disable the entire crew: it is transient and requires little intervention. The risks must be interpreted differently. In addition, correlations between deficits play a key role in risk estimates. Unassisted crew egress from a spacecraft is a telling example. Assume that loss of aerobic capacity (Moore et al., 2014) and muscle strength (Ryder et al., 2013) is associated with neurovestibular deficits on an individual basis. A small crew returning from Mars might have two members incapacitated from sensorimotor orientation disturbances, and associated deficits in aerobic and muscle capacity. In this case, the remaining crew who have the strength and are not suffering from disorientation or nausea can come to their aid. If, on the other hand, those who have problems with spatial orientation or motion sickness are different from those who are weakest and have less capacity to help their crewmates, then the entire crew is at increased risk. Thus, understanding correlations between the different physiological impairments due to space flight—if in fact there are correlations—is not only an interesting scientific question but also one of great practical import for mission design and analysis (Jennings et al., 1988; White and Averner, 2001).

## Countermeasures

Just as balance, orientation, and spatially appropriate responses rely on multiple sensory receptors and motor effectors (e.g., Maurer et al., 2006), so too will successful sensorimotor countermeasures likely need to be multivariate. The case of unassisted egress, for example, will possibly need a combination of head movements during reentry (to begin acclimatization to a new pattern of otolith stimulation: Paloski et al., 2008), training for strong visual dependence (to orient properly to the spacecraft interior: Oman, 2003), a vest to provide vibrotactile orientation or direction cues (Sienko et al., 2013), and some form of adaptive generalization or contextualization (Shelhamer and Zee, 2003; Mulavara et al., 2009). An intriguing line of investigation is suggested by the finding that locomotion in the face of vestibular damage can sometimes be carried out if it is rapid and ballistic rather than slow and methodical; the former case presumably not relying on missing or inaccurate vestibular information (Brandt, 2000). This raises the possibility of a countermeasure that involves training of crew to make ballistic movements and not to depend on unreliable sensory information. This could be very effective in an emergency excursion to get away from a damaged vehicle, but it is not as clear how to implement in the course of piloting or egress. It should be noted, however, that pilots learn to ignore unreliable vestibular orientation cues and rely on their cockpit instruments when acquiring advance piloting skills.

There are more broad-based countermeasures which are geared toward maintaining the spectrum of overall function in different environments (one key environmental factor being tonic g level). These include adaptive generalization (Mulavara et al., 2009) and contextual adaptation (Shelhamer and Zee, 2003). Both of these suffer from the fundamental problem of needing to provide a tonic otolith cue (to mimic the gravity environment of a planetary surface) while in the weightlessness of space. The effectiveness of either of these would be increased if there was a way to provide otolith stimulation in flight that at least approximated that which occurs during head movements in a gravity field, in which case the maintenance of an otolith-mediated tilt response would at least have meaning in flight. A prophylactic vestibular prosthesis (Merfeld and Lewis, 2012) is an extreme solution to this problem, which could provide the missing “tilt” stimulation to the vestibular afferents during “tilt” (relative to visual upright) in space.

## Interactions

More broadly, the key relevance of sensorimotor concerns may come in their interactions with other disciplines. Here, the maturity of the field can be used to advantage, in that the basic aspects of neurovestibular function are well characterized (Goldberg et al., 2012), making it easier (though far from trivial) to begin investigating interactions with other systems. Even a cursory review of the literature will show that the sensorimotor and neurovestibular systems have an impact on a large variety of other systems.

The relationship between vestibular deficits and cognitive effects is one area of obvious interaction. Is it clear from patients with vestibular disorders that mood is affected (Bigelow et al., 2015). More subtly, however, there are certainly contributions of vestibular information to spatial orientation and memory, and to motor reflexes (Smith and Zheng, 2013; Palla and Lenggenhager, 2014). Disruption of this input could be a main source of “space fog” or the “space stupids” experienced by some crew members: the perceived moderate slowing of cognitive function early in flight (Kanas and Manzey, 2008). It is not hard to imagine that extra mental effort is needed to perform many mundane tasks when usually reliable cues for orientation and reflex control are altered. A related phenomenon is seen in people with compromised balance function who have difficulty with dual-tasking since otherwise excess mental effort must be devoted to normally automatic processes such as maintaining balance while walking (Springer et al., 2006).

As another example, with current exercise countermeasures, muscle deconditioning after extended space flight is not the problem that it used to be, due to improved devices and protocols that involve intervals of high-intensity exercise (Ploutz-Snyder et al., 2014). Crew members return with generally good strength, but they are still impaired in their abilities to coordinate these muscles for locomotion (Cohen et al., 2012). The tonic influence of vestibular pathways undoubtedly plays a major role in this function, a role which is altered as a consequence of space flight (Cohen et al., 2012).

Relatively little studied are the impacts of vestibular and sensorimotor deficits on behavioral and interpersonal issues. Bodily self-perception can be altered with vestibular pathology (Lopez, 2013), and this altered sense of self-and-other can impact relationships with others and with the environment (Deroualle and Lopez, 2014). Given concerns about team cohesion and the potential for interpersonal conflicts on extended flights, this is an area worth investigating (Salas et al., 2015).

Perhaps the most well-known of vestibular-mediated responses outside of the CNS is that of vestibulo-autonomic interactions (Yates et al., 2014), which can have a significant direct impact on orthostatic intolerance as well as more diffuse tonic responses. Less well appreciated are findings that vestibular lesions in rats produce loss in the weight-bearing bones, associated with increased sympathetic activation (Vignaux et al., 2013). Given the concerns about loss of bone density and strength in extended space flight (Smith et al., 2012), these types of interactions deserve more scrutiny.

These widespread inter-disciplinary connections have interesting conceptual and practical implications. Conceptually, one might conceive of the body as a complex network composed of a large number of highly interconnected subsystems. Depending on the specific topology, such a network might have a few key critical nodes or hubs. The neurovestibular system might be such a hub, given its widespread connections to other systems. This leads to the hope that one might understand these interacting influences by better understanding the interconnections and the emergent properties of mathematical networks with these properties (Newman, 2003). This conceptual structure has practical import, as it suggests that attacking a central hub with an appropriate countermeasure might have widespread consequences. In other words, a countermeasure that maintains or restores some semblance of normal vestibular function might also serve to mitigate problems associated with cognition, mood, bone loss, and cardiovascular function. This is especially appealing in that such a networked countermeasure might make more efficient use of limited in-flight resources than a set of countermeasures each dedicated to a separate physiological subsystem.

## Analogs

In-flight resources such as crew time will always be in limited supply for research purposes. Therefore, most space programs make use of ground-based analog facilities whenever possible (Palinkas et al., 2000; Meck et al., 2009; Crucian et al., 2014). Analogs reproduce some aspects of the flight environment, such as fluid shifts and physiological deconditioning with head-down bed rest, and isolation and confinement in extreme environments as in Antarctic winter-overs. Analogs have been extremely valuable for the study of many problems in a less-expensive and better-controlled setting than space flight. We do not yet, however, have a good analog for the sensorimotor disturbances of prolonged space flight which could be used in ground studies for the development of countermeasures. This is especially true regarding the vestibular system and otolith effects. While the unloading due to extended bed rest can reproduce some ascending effects due to proprioceptive and other changes (Reschke et al., 2009), altered descending influences are more difficult to recreate. By descending effects we mean those effects on posture, locomotion, and other aspects of motor control that are mediated by vestibular circuitry and processing. Ascending effects are due to reciprocal sensory information from proprioceptors and other somatic graviceptors, which flows from body to brain. Galvanic vestibular stimulation (GVS), for example, causes disruptions that might provide a basis for such a procedure, but the mechanisms and effects are diffuse (Wardman and Fitzpatrick, 2002; Moore et al., 2011). Likewise, transcranial magnetic stimulation (TMS) can disrupt some aspects of spatial orientation (Kheradmand et al., 2015) but does not act at the level of the otolith afferents or end organ. A form of functional ablation of otolith activity would be useful, but even here the results would miss the fact that the otolith organs are still active in space and accurately transduce those linear accelerations that do not involve tilt. Barring the development of such an analog, it is conceivable to perform many types of sensorimotor research in spaceflight settings other than International Space Station (ISS). Since g transitions are a major problem, shorter flights on commercial vehicles (which will soon provide crew delivery and return service to ISS) can help fill the gap.

## Conclusion

The research community has made great progress on many of the key issues involving sensorimotor—especially neurovestibular—disturbances in the spaceflight setting. While many questions remain to be settled, it is unlikely that sensorimotor problems will be among the greatest risks to crews on long-duration space flights. However, this does not mean that sensorimotor issues are irrelevant. Quite the contrary: due to the widespread interactions of neural processes involved in sensorimotor control with many other physiological systems, increased understanding of the sensorimotor system can help to address not only purely sensorimotor concerns but also contribute to the solution of problems in many other disciplines. This can also be a key step in promoting integrative physiology and the breaking down of disciplinary barriers between physiological systems. Better integration not only reflects the reality of the organism (White and Averner, 2001) but might enable the development of integrated countermeasures that address multiple deficits in an efficient manner.

## Conflict of Interest Statement

The Associate Editor, Dr Ajitkumar Mulavara and the Review Editor, Dr Angus Rupert declare that, despite being affiliated with the National Space Biomedical Research Institute (NSBRI), the review process was handled objectively. The author declares that the research was conducted in the absence of any commercial or financial relationships that could be construed as a potential conflict of interest.
